# Effects of Tauroursodeoxycholic Acid and 4-Phenylbutyric Acid on Selenium Distribution in Mice Model with Type 1 Diabetes

**DOI:** 10.1007/s12011-022-03193-8

**Published:** 2022-03-18

**Authors:** Dongyang Xing, Qi Zhou, Yiting Wang, Jiancheng Xu

**Affiliations:** 1 Department of Laboratory Medicine, First Hospital of Jilin University, 1 Xinmin Street, Changchun, 130021 China; 2 Department of Pediatrics, First Hospital of Jilin University, 1 Xinmin Street, Changchun, 130021 China

**Keywords:** Selenium, Tauroursodeoxycholic acid, Type 1 diabetes, 4-Phenylbutyric acid

## Abstract

The effect of selenium on diabetes is significant. As pharmaceutical chaperones, tauroursodeoxycholic acid (TUDCA) and 4-phenylbutyric acid (4-PBA) can effectively improve the oxidative stress of the endoplasmic reticulum. This study established a mice model with type 1 diabetes (T1D) to evaluate the effects of pharmaceutical chaperones on selenium distribution. Streptozotocin was used to induce Friend virus B-type mice to establish a T1D mice model. Mice were administered with TUDCA or 4-PBA. Selenium levels in different tissues were measured by inductively coupled plasma-mass spectroscopy (ICP-MS). After treatment with TUDCA and 4-PBA, related laboratory findings such as glucose and glycated serum protein were significantly reduced and were closer to normal levels. At 2 weeks, 4-PBA normalized selenium levels in the heart, and 4-PBA and TUDCA maintained the selenium in the liver, kidney, and muscle at normal. At 2 months, 4-PBA and TUDCA maintained the selenium in the heart, liver, and kidney at normal levels. The serum selenium had a positive correlation with zinc and copper in the diabetes group and the control group, while the serum selenium had no significant association with magnesium and calcium at 2 weeks and 2 months. TUDCA and 4-PBA have crucial effects on selenium distribution in diabetic mice, and further research is needed to research their internal mechanisms.

## Introduction

Type 1 diabetes (T1D) is usually a metabolic disease caused by autoimmune damage, also known as insulin-dependent diabetes. During the progression of T1D, it can cause serious complications such as cardiovascular and cerebrovascular disease, kidney disease, and neuropathy. Some studies show that fluctuation of trace elements and elevated oxidative stress may cause insulin dysfunction, diabetes, and diabetes complications [1]. So if T1D is not well controlled, blood sugar will increase the severity of oxidative stress and cause more diabetes complications. Therefore, dietary intake requirements are more stringent for diabetics. In the daily diet, the intake of trace elements is essential for the development of diabetes. At the same time, the progress of diabetes will also affect the metabolic balance of trace elements in the body [2].

As one of the necessary trace elements, selenium is an essential component of selenoproteins, which are involved in anti-oxidation, anti-inflammatory, and thyroid hormone metabolism [3]. The discussion of the relationship between selenium and diabetes has become a hot topic in recent years but is controversial. In fact, the relationship between the two is particularly complex, so many studies have reached different views. Studies have shown that selenium can help prevent and treat diabetes via antioxidant and anti-oxidative stress effects [4, 5]. However, the results of the National Health and Nutrition Examination Survey [6] and observational studies [3–7] indicate that serum selenium levels have positive correlations with the onset of diabetes mellitus.

Social progress and changes in dietary habits have increased the risk of lifestyle diseases, including hypertension, diabetes, and obesity [8]. Most of these diseases are associated with endoplasmic reticulum stress and the impairment of protein folding mechanisms [9]. Therefore, “pharmaceutical chaperones,” including tauroursodeoxycholic acid (TUDCA) and 4-phenylbutyric acid (4-PBA), can potentially reduce endoplasmic reticulum stress. TUDCA, a taurine conjugate of ursodeoxycholic acid, is used in the treatment of primary cholangitis based on its bile activity and ability to protect hepatocytes [10]. Numerous studies have demonstrated that the activity of TUDCA can be applied to areas other than hepatobiliary diseases, including diabetes, obesity, cardiovascular disease, or cancer [11]. The loss and dysfunction of pancreatic β cells can lead to T1D [12]. Expression defects of unfolded protein response regulator are found in β cells in human and mice models of T1D [12]. TUDCA can restore the expression of unfolded protein response modulators, decreasing β cell apoptosis, maintaining insulin secretion, and reducing blood glucose [12, 13]. 4-PBA, a low molecular weight fatty acid, mitigates cellular damage by reducing endoplasmic reticulum stress. 4-PBA enhanced the folding capacity of the endoplasmic reticulum and attenuated endoplasmic reticulum stress in diabetic rats [14]. Studies have also shown that the treatment of 4-PBA could efficaciously repress the progression of nephropathy in diabetic mice induced by adjusting the endoplasmic oxidative reticulum stress [15, 16].

Our previous studies demonstrated that TUDCA and 4-PBA have affected the distribution of calcium, magnesium, copper, and zinc in T1D mice model [17, 18]. However, few studies have assessed the influence of TUDCA and 4-PBA on selenium metabolism in diabetes. This study assessed the effect of TUDCA and 4-PBA on selenium distribution by measuring selenium levels in T1D mice model different tissues.

## Materials and Methods

### T1D Mice Model

The 8-week-old male Friend virus B-type (FVB) mice were obtained from Vital River Laboratories (Beijing, China) and were fed in the Experimental Animal Center in the College of Basic Medical Sciences, Jilin University, at 22 °C under a 12-h light/12-h dark cycle, with free access to rodent food and water. This experiment was approved by the Ethics Committee of Jilin University First Hospital.

The mice were randomly divided into six experimental groups, namely, non-diabetic control mice (CM); control mice administered with 4-PBA for non-diabetic (4-PBA); and control mice administered with TUDCA for non-diabetic (TUDCA); mice administered with 4-PBA for diabetic (DM + 4-PBA); mice administered with TUDCA for diabetic (DM + TUDCA); diabetic mice (DM). Mice in all groups were fed with standard laboratory chow (Mouse Feed Food, No. 8061, Chengdu Dashuo laboratory animal Co., Ltd., Chengdu, China), which contained 18% protein, 4% fat, 5% fiber, 10% ash content, 1.4% Se.

Mice were made T1D diabetic model by intraperitoneal injection of multiple, low-dose streptozotocin (STZ; Sigma Chemical Co., St. Louis, MO, USA; 40 mg/kg, dissolved in sodium citrate buffer, pH = 4.5). When the last injection of STZ is finished, a blood glucose meter was used (Bayer HealthCare, Mishawaka, IN, USA) to measure the blood glucose of mice tail vein. Fasting blood glucose > 12 mmol/L after STZ treatment in T1D mice are considered diabetic [19]. Two days before stopping STZ injection, phosphate buffered saline was used daily to make the mice adapt to the environment. All groups used for 4-PBA treatments received 100 µL PBS twice per day at 8 o’clock in the morning and afternoon by gavage and the groups used for TUDCA treatments received intraperitoneal injection of 100 µL PBS at the same point in time for 3 consecutive days. When the last injection of STZ is finished, fasting blood glucose levels were measured from the mice tail vein at 8 am and the treatments of 4-PBA and TUDCA were started (Day 0). 4-PBA (Merck KGaA, Hohenbrunn, Germany) was given with two separated dosages (500 mg/kg at 8 o’clock in the morning and afternoon, 1 g/kg/day) through oral gavage. Same as 4-PBA, TUDCA (Calbiochem, La Jolla, CA, USA) was intraperitoneally injected (250 mg/kg at 8 o’clock in the morning and afternoon, 500 mg/kg/day). The DM + 4-PBA and DM + TUDCA group was injected the same volume of carrier fluid intragastrically or intraperitoneally, respectively. Body weight and fasting blood glucose were detected routinely.

Mice administered with 4-PBA and TUDCA at 2 weeks and 2 months were sacrificed intraperitoneally with 2% pentobarbital sodium (30 mg/kg) and heart puncture. All tissues were collected and preserved at − 80 °C. Whole blood specimens from the retro-orbital plexus were placed in metal-free containers and were centrifuged at 12000r/min for 5 min at 4 °C. The supernatant liquid was added to the test tube, frozen with shock, and kept at − 80 °C for further examination.

### Selenium Detection

Approximately 0.5–1 g of every tissue was required for experiment in 1 mL nitric acid separate at indoor temperature and heated to 110 °C for 8 h to promote digestion. Then, 4 mL deionized water was added, and the obtained solution was tested. Selenium level was measured using inductively coupled plasma-mass spectroscopy (ICP-MS, Agilent Technologies, Santa Clara, CA, USA). The equipment parameters are adjusted daily to assure accuracy. The ICP-MS system was operated at 1550 W radio frequency power and 1.05 L/min nebulizer gas flow rate. Selenium levels were showed as micrograms per liter wet tissue.

### Testing of Other Laboratory Findings

Glycated serum protein (GSP), blood urea nitrogen (BUN), creatinine (Cre), uric acid (UA), total cholesterol (TC) riglyceride (TG), high-density lipoprotein (HDL), and low-density lipoprotein cholesterol (LDL) levels were measured by Hitachi 7600–010 Clinical Chemistry Analyzer (Hitachi, Tokyo, Japan).Periodic maintenance, functional checks, calibration, quality control, and procedures were carried out for the assay manufacture using the instructions equipment.

### Statistical Analysis

Indicators in the basic information are represented by Mean ± SE. Comparison between different groups by Student’s *t* test. The *p* values were two-sided (*P* < 0.05, statistically different). SPSS version 26.0 and Graphpad Prism version 8.0 were used for statistical analyses.

## Results

### Basic Information of T1D Mice

**Fig. 1:**
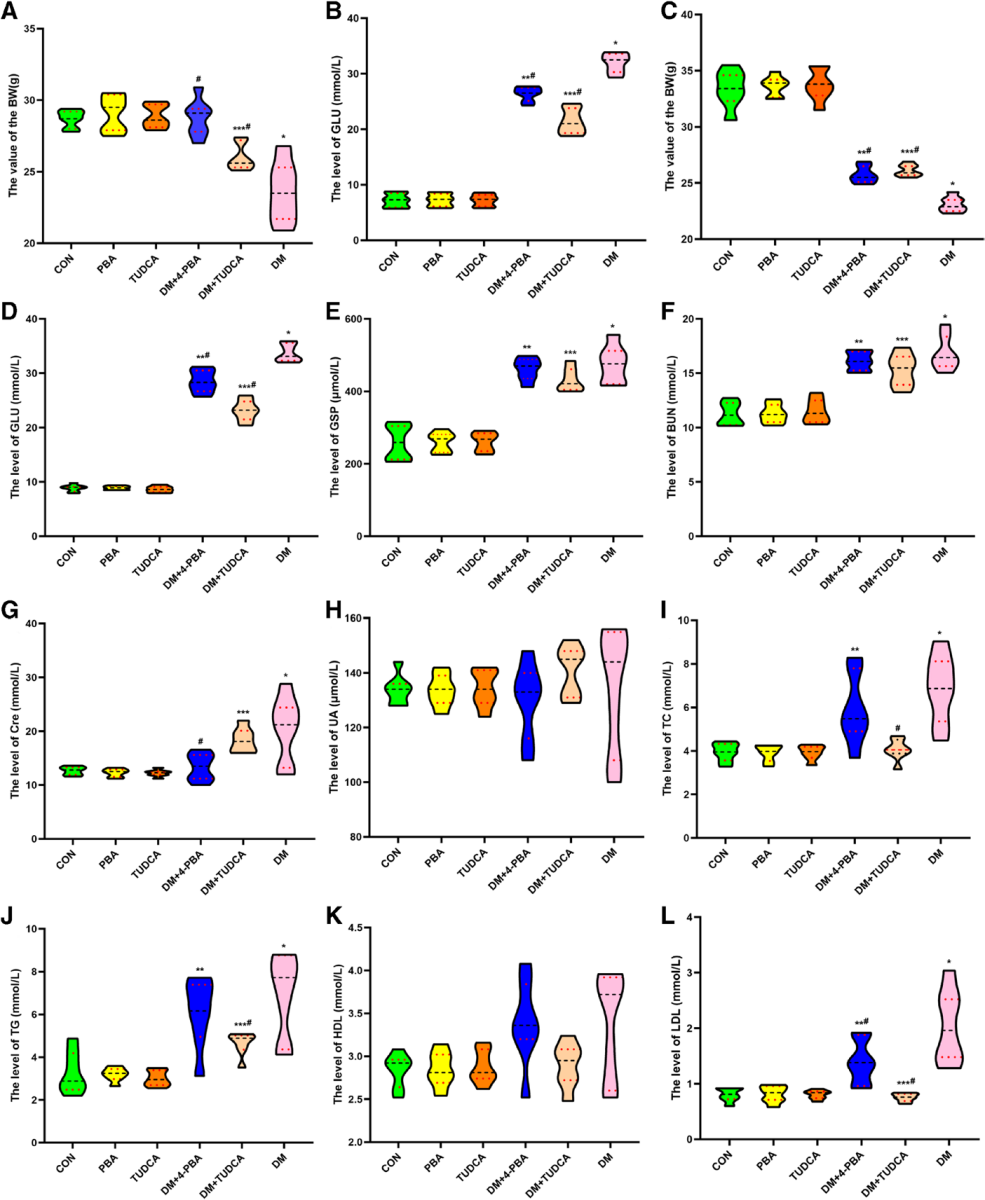
**A**–**L** Basic information of T1D mice in CM, 4-PBA, TUDCA, DM + 4-PBA, and DM + TUDCA groups at 2 WAT and 2 MAT. **A**–**B** represent the T1D mice BW and Glu at 2 WAT; **C**–**L** represent the T1D mice BW, Glu, GSP, BUN, Cre, UA, TC, TG, HDL, LDL at 2 MAT. GSP, glycated serum protein; BUN, blood urea nitrogen; Cre, creatinine; UA, uric acid; TC, total cholesterol; TG, triglyceride; HDL, high-density lipoprotein; LDL, low-density lipoprotein cholesterol. Data are presented as mean ± SE, *n* = 7 in all groups. CM non-diabetic control mice, DM diabetic mice group, DM + 4-PBA group of diabetic mice treated with PBA, DM + TUDCA group of diabetic mice treated with TUDCA, 4-PBA group of non-diabetic control mice treated with 4-PBA, TUDCA group of non-diabetic control mice treated with TUDCA. *P < 0.05 vs. CM group; **P < 0.05 vs. 4-PBA group; ***P < 0.05 vs. TUDCA group; ^#^*P* < 0.05 vs. DM group

The basic information of T1D mice administered with TUDCA and 4-PBA is shown in Table 1 and Fig. 1A–L. The mean value of the body weight of the groups CM, 4-PBA, TUDCA, DM + 4-PBA, DM + TUDCA, and DM was as follows: 2 weeks after treatment (WAT): 28.7, 29.1, 28.9, 28.8, 26.0, and 23.6 (g); 2 months after treatment (MAT): 33.4, 33.6, 33.7, 25.8, 26.1, and 23.1 (g). DM had a lower body weight (*P* < 0.05) than CM, and the DM + 4-PBA and DM + TUDCA had higher body weight than DM (*P* < 0.05). The mean value of the Glu level in the CM, 4-PBA, TUDCA, DM + 4-PBA, DM + TUDCA, and DM group was as follows: 2 WAT: 7.2, 7.2, 7.2, 26.4, 21.4, and 31.9 (g); 2 MAT: 8.8, 8.9, 8.6, 28.5, 23.1, and 33.7 (g). The DM had more reduced Glu levels than CM (*P* < 0.05), and the DM + 4-PBA and DM + TUDCA had lower Glu levels than DM (*P* < 0.05).

**Table 1:** Determination of serum selenium and other elements in type 1 diabetic mice

	2 weeks						2 months					
	CM	4-PBA	TUDCA	DM + 4-PBA	DM + TUDCA	DM	CM	4-PBA	TUDCA	DM + 4-PBA	DM + TUDCA	DM
S-Se (mg/L)	0.57 ± 0.02	0.56 ± 0.02	0.52 ± 0.02	0.70 ± 0.02**	0.71 ± 0.02***^#^	0.75 ± 0.04*	0.51 ± 0.03	0.52 ± 0.02	0.51 ± 0.03	0.70 ± 0.01**	0.75 ± 0.04***^#^	0.72 ± 0.06*
Cu (mg/L)	1.08 ± 0.74	1.09 ± 0.76	1.10 ± 0.18	1.34 ± 0.12	1.37 ± 0.37	1.39 ± 0.70	1.08 ± 0.62	1.09 ± 0.60	1.08 ± 0.52	1.04 ± 0.31	1.37 ± 0.34	1.42 ± 0.12
Zn (mg/L)	1.48 ± 0.45	1.48 ± 0.43	1.48 ± 0.43	1.43 ± 0.41	1.38 ± 0.30	1.37 ± 0.31	1.40 ± 0.45	1.41 ± 0.44	1.40 ± 0.48	1.43 ± 0.41	1.42 ± 0.32	1.26 ± 0.60
Mg (mg/L)	38.9 ± 0.95	39.5 ± 1.03	39.8 ± 1.16	34.5 ± 1.10	40.9 ± 0.79	33.5 ± 0.89	38.7 ± 1.75	38.4 ± 0.47	38.7 ± 1.61	35.4 ± 0.86	41.3 ± 1.39	33.7 ± 0.30
Ca (mg/L)	111.3 ± 3.4	111.9 ± 3.1	111.5 ± 3.6	93.0 ± 2.0	122.6 ± 2.1	91.1 ± 2.8	106.5 ± 2.4	106.8 ± 2.4	110.9 ± 2.5	96.5 ± 3.0	104.2 ± 2.5	95.4 ± 2.5

Data are expressed as mean ± SE, *n* = 7 in all groups

^*^P < 0.05 vs. CM group; **P < 0.05 vs. 4-PBA group; ***P < 0.05 vs. TUDCA group; ^#^*P* < 0.05 vs. DM group

*BW*, body weight; *Glu*, serum glucose; *GSP*, glycosylated serum protein; *BUN*, blood urea nitrogen; *Cre*, creatinine; *Mg*, magnesium; *Ca*, calcium; *TC*, total cholesterol; *TG*, triglyceride; *HDL*, high-density lipoprotein cholesterol; *LDL*, low-density lipoprotein

CM non-diabetic control mice, DM diabetic mice group, DM + 4-PBA group of diabetic mice treated with PBA, DM + TUDCA group of diabetic mice treated with TUDCA, 4-PBA group of non-diabetic control mice treated with 4-PBA, TUDCA group of non-diabetic control mice treated with TUDCA

At 2 MAT, the mean value of laboratory findings in CM, 4-PBA, TUDCA, DM + 4-PBA, DM + TUDCA, and DM group were as follows: GSP (μmol/L): 259.6, 260.3, 261.1, 460.6, 431.7, and 475.4; BUN (mmol/L): 11.3, 11.3, 11.5, 16.1, 15.3, and 16.8; Cre(mmol/L): 12.7, 12.3, 12.2, 13.3, 18.4, and 20.5; UA(μmol/L): 133.9, 133.6, 134.3, 130.0, 140.9, and 134.3; TC(mmol/L): 3.9, 3.9, 3.9, 5.9, 4.1, and 6.9; TG (mmol/L): 3.3, 3.2, 3.0, 6.1, 4.6, and 6.9; HDL (mmol/L): 2.8, 2.8, 2.9, 3.4, 2.9, and 3.4; LDL (mmol/L): 0.81, 0.81, 0.81, 1.39, 0.76, and 2.04. Compared with the CM, the GSP and BUN levels in DM + 4-PBA, DM + TUDCA, and DM were obviously elevated (*P* < 0.05), and the Cre, TC, TG, LDL, in DM were obviously higher (*P* < 0.05). Compared with the DM, the Cre levels in DM + 4-PBA were more reduced (*P* < 0.05), the TC and TG levels in DM + TUDCA were lower (*P* < 0.05), and the LDL levels in DM + 4-PBA and DM + TUDCA were more reduced (*P* < 0.05).

### Selenium Level in Every Tissue at Different Periods

**Fig. 2:**
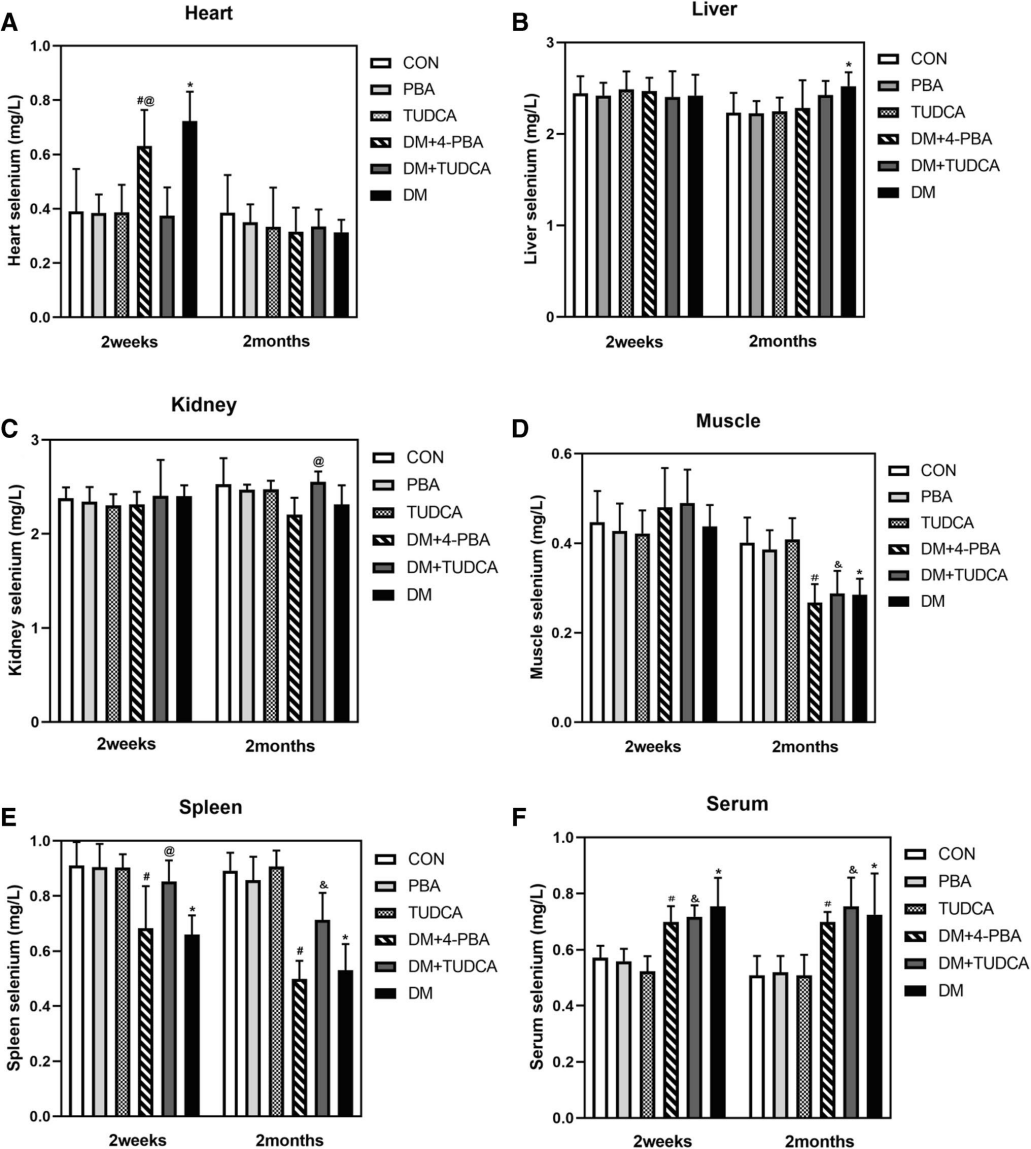
**A**–**F** represent selenium levels in the heart, liver, kidney, muscle, spleen, and serum of T1D mice at 2 WAT versus 2 MAT. Data are presented as mean ± SE, *n* = 7 in all groups. CM non-diabetic control mice, DM diabetic mice group, DM + 4-PBA group of diabetic mice treated with PBA, DM + TUDCA group of diabetic mice treated with TUDCA, 4-PBA group of non-diabetic control mice treated with 4-PBA, TUDCA group of non-diabetic control mice treated with TUDCA. *P < 0.05 vs. CM group; ^#^*P* < 0.05 vs. 4-PBA group; ^&^*P* < 0.05 vs. TUDCA group; ^@^*P* < 0.05 vs. DM group

Selenium levels in different organs of T1D mice model are shown in Fig. 2A–F. At 2 WAT, the heart selenium levels in DM were more higher than that in DM + 4-PBA, DM + TUDCA, and CM, respectively. Compared with 4-PBA, the heart selenium level in DM + 4-PBA was significantly more higher at 2 MAT. Compared with CM, the liver selenium levels in DM were slightly evaluated at 2 MAT. Compared with DM + 4-PBA, the kidney selenium levels in DM were higher at 2 WAT. Compared with DM + TUDCA, the kidney selenium levels in DM were lower at 2 MAT. From 2 WAT to 2 MAT, muscle selenium level was reduced in DM + 4-PBA, DM + TUDCA, and DM groups. At 2 MAT, the muscle selenium levels in DM + 4-PBA, DM + TUDCA, and DM were lower than that in their corresponding control groups, respectively. Compared with PBA, the spleen selenium levels in DM + 4-PBA were reduced at 2 WAT. Compared with DM, the spleen selenium levels in CM and DM + TUDCA were more higher at 2 MAT. At 2 WAT and 2 MAT, the serum selenium levels of DM + 4-PBA, DM + TUDCA, and DM were more obviously evaluated than that in their corresponding control groups.

### Correlation Between Serum Levels of Selenium and Serum Copper, Zinc, Calcium, and Magnesium Levels

The relationship of serum selenium level with copper, zinc, calcium, and magnesium levels was analyzed (Tables 2 and 3). At 2 WAT, serum selenium levels in DM + 4-PBA, DM + TUDCA, and DM were significantly positively correlated with copper and zinc levels. However, there was no significant association between selenium, magnesium, and calcium levels in the study groups, except in DM. At 2 MAT, selenium levels in DM + TUDCA and DM had significantly positive correlation with copper and zinc, but not with magnesium and calcium.

**Table 2:** Association of Se and other trace elements in serum of diabetic mice treated with PBA or TUDCA for 2 weeks

	Se											
	CM		4-PBA		TUDCA		DM + 4-PBA		DM + TUDCA		DM	
	*r*	*p*	*r*	*p*	*r*	*p*	*r*	*p*	*r*	*p*	*r*	*p*
Cu	0.605	0.150	0.917	0.004*	0.949	0.001*	0.903	0.005*	0.833	0.020*	0.986	< 0.001*
Zn	0.745	0.055	0.908	0.005*	0.899	0.006*	0.935	0.002*	0.880	0.009*	0.851	0.015*
Mg	0.167	0.721	0.818	0.024*	0.763	0.046*	0.544	0.207	0.064	0.892	-0.658	0.108*
Ca	0.156	0.739	0.872	0.011*	0.908	0.005*	0.222	0.632	-0.131	0.780	-0.760	0.047*

Abbreviations’ spellings are same as the description for Table 1

^*^P < 0.05 for the association

**Table 3:** Association of Se and other trace elements in serum of diabetic mice treated with PBA or TUDCA for 2 months

	Se											
	CM		4-PBA		TUDCA		DM + 4-PBA		DM + TUDCA		DM	
	*r*	*p*	*r*	*p*	*r*	*p*	*r*	*p*	*r*	*p*	*r*	*p*
Cu	0.816	0.025*	0.827	0.022*	0.963	0.001*	0.546	0.205	0.877	0.009*	0.937	0.002*
Zn	0.907	0.005*	0.949	0.001*	0.959	0.001*	0.606	0.149	0.947	0.001*	0.985	< 0.001*
Mg	0.631	0.128	0.889	0.007*	0.819	0.024*	0.561	0.191	-0.061	0.896	0.195	0.675
Ca	0.731	0.062	0.888	0.008*	0.995	< 0.001*	0.868	0.011*	0.442	0.321	0.053	0.910

Abbreviations’ spellings are same as the description for Table 1

^*^P < 0.05 for the association

## Discussion

4-PBA and TUDCA treatment affected the body weight and laboratory results of T1D mice. At 2 WAT and 2 MAT, body weight decreased and blood glucose increased. 4-PBA and TUDCA alleviated the changes of blood glucose and body weight, and regulated the levels of TC, TG, GSP, BUN, LDL, and Cre to close to the normal level.

### Selenium Levels in Organs and Serum

This study established a T1D mice model to explore the influence of 4-PBA and TUDCA treatment on selenium distribution in above tissues. At 2 WAT and 2 MAT, selenium levels increased in the serum and decreased in the heart, muscle, and spleen of the DM + 4-PBA group. In the DM + TUDCA group, selenium levels increased in the kidney, liver, and serum and decreased in the muscle and spleen. There was no significant difference in selenium levels in the liver and kidneys among the groups. The levels of selenium in different tissues are different. In another study on camel, selenium levels were higher in the kidney and liver [20], which was consistent with our study.

Selenium is a kind of antioxidant stress functions of trace elements. Lack of the selenium in the human body susceptible to oxidative stress damage [21]. It has been reported that selenium can control the progression of diabetes due to its antioxidant properties [22]. Selenate also has insulin-like effects in type 2 diabetes (T2D) animals and improves insulin resistance [23]. Other studies mentioned that both small and large amounts of selenium have been found to severely affect the cardiovascular system [24, 25]. The association between excess selenium intake and cardiac dysfunction may be due to oxidative stress and complications of diabetes [26]. In this study, the heart selenium content of T1D mice was significantly increased at 2 weeks, which also verified that the increase in heart selenium mentioned in previous studies can cause myocardial disease [27]. Selenium regulates the activity of glucose-metabolizing enzymes in diabetes and improves glucose absorption and metabolism in the liver [28]. Cristina [29] et al. found that selenium concentrations increased in the liver, kidney, and spleen of diabetic individuals. Accordingly, our results confirmed that selenium concentrations increased in the liver and decreased in the kidney and spleen. Li [30] et al. confirmed that the lack of selenium in the spleen could directly lead to oxidative stress. From this study, it can be seen that the selenium level of T1D mice after TUDCA treatment was significantly evaluated. Therefore, maintaining the stability of selenium levels in tissues and serum will have a certain impact in the treatment of diabetes.

### Correlation Between the Serum Levels of Selenium and Other Trace Elements

This study evaluated the correlation between serum selenium and other trace elements in mice. At 2 WAT and 2 MAT, selenium was strongly correlated with copper and zinc levels. These results agree with our previous study, in which serum selenium was not correlated with magnesium and calcium [31]. As an important trace element in human, copper participates in different biological processes, including mitochondrial respiration and antioxidant defense [32]. It has been shown that copper nanoparticles have antioxidant and free radical scavenging ability and improve cardiovascular function in diabetic mice [33]. As a trace element, zinc participates in cell division, immune response, and the control of insulin secretion. Studies have shown that zinc nanoparticles improve serum glucose and insulin levels and glucose tolerance in T1D and T2D mice [34].

Few studies have assessed the correlation between copper, zinc, and selenium. This study proved that selenium had an obvious positive correlation with copper and zinc levels but not with magnesium and calcium levels. Assimina reported that serum magnesium levels of T1D children or adolescents were lower than the control group, which was same with the results of our study [35]. This finding may be related to the increased excretion of magnesium in urine, retention of magnesium by insulin in distal tubules, or insufficient intake of magnesium [35]. Previous studies have shown that L-type calcium channels are participated in cardiomyocytes injury caused by selenium deficiency and are positively correlated with oxidative stress. Moreover, selenium deficiency can cause calcium overload [36]. To our knowledge, no studies have evaluated the correlation between selenium, magnesium, and calcium levels.

### The Effect of TUDCA and 4-PBA on Selenium Distribution

Small molecule pharmaceutical chaperones, such as TUDCA and 4-PBA, can reduce endoplasmic reticulum stress by improving the folding ability of endoplasmic reticulum proteins, controlling insulin resistance and T2D [37]. TUDCA is the main active ingredient of bear bile, which is mainly used to treat primary biliary cirrhosis and chronic hepatitis [38]. 4-PBA is an ammonia scavenger approved by the US Food and Drug Administration to treat urea cycle disorders. Furthermore, 4-PBA has been used in clinical trials to treat other diseases, such as cystic fibrosis and thalassemia [37].

Studies have shown that TUDCA and 4-PBA can enhance the effect of insulin and reduce blood sugar levels by regulating endoplasmic reticulum stress, and have potential in the treatment of diabetes [39, 40]. In this study, at 2 WAT and 2 MAT, 4-PBA and TUDCA normalized selenium level in the heart, and maintained selenium in the liver and kidneys at normal levels. Our research group previously explored the effects of TUDCA and 4-PBA on copper, zinc, calcium, and magnesium in T1D mice. 4-PBA normalized kidney magnesium and muscle calcium levels, and TUDCA normalized magnesium levels in the liver, kidneys, and serum and calcium levels in the kidneys and serum [18]. In the liver of DM, 4-PBA fully normalized zinc levels at 2 WAT and partially normalized these levels at 2 MAT [17]. These results show that pharmaceutical chaperones enhance the beneficial effects of trace elements on T1D. Moreover, TUDCA and 4-PBA differentially regulated selenium levels in different tissues. Therefore, the regulatory mechanism of pharmaceutical chaperones on the homeostasis of trace elements in T1D mice needs to be further studied.

## Conclusion

Changes in the levels of trace elements in diabetic patients have attracted widespread attention. TUDCA and 4-PBA improve insulin resistance and blood glucose levels. This study measured selenium levels in tissues and serum of T1D mice treated with TUDCA and 4-PBA. The results showed that (1) selenium levels in the heart, muscle, spleen, and serum of T1D mice were significantly different from those of the control group; (2) selenium levels in T1D mice were positively correlated with copper and zinc levels, but it was not significantly correlated with calcium and magnesium levels at 2 WAT and 2MAT; (3) at 2 WAT, selenium levels increased in the heart and serum but decreased in the spleen of the DM + 4-PBA group. Selenium increased in the serum of the DM + TUDCA group. At 2 MAT, 4-PBA and TUDCA normalized selenium levels in the heart, liver, and kidneys. The average selenium levels decreased in muscles and spleen and increased in the serum. In summary, TUDCA and 4-PBA regulated the levels of trace elements in T1D mice, demonstrating that pharmaceutical chaperones can potentially treat diabetes. At present, few studies evaluated the effects of TUDCA and 4-PBA on selenium levels in diabetic mice or patients, and further research is needed to determine the mechanisms by which chaperones regulate the levels of trace elements. This study provided clues for the diagnosis and treatment of trace elements in clinical diabetic patients.

## Data Availability

The datasets generated during and/or analyzed during the current study are not publicly available due to privacy concerns but are available from the corresponding author on reasonable request.
