# Correction to: Effects of Tauroursodeoxycholic Acid and 4-Phenylbutyric Acid on Selenium Distribution in Mice Model with Type 1 Diabetes

**DOI:** 10.1007/s12011-023-03572-9

**Published:** 2023-01-14

**Authors:** Dongyang Xing, Qi Zhou, Yiting Wang, Jiancheng Xu

**Affiliations:** 1 Department of Laboratory Medicine, First Hospital of Jilin University, 1 Xinmin Street, Changchun, 130021 China; 2 Department of Pediatrics, First Hospital of Jilin University, Changchun, 130021 China

**Correction to: Biological Trace Element Research**

**https://doi.org/10.1007/s12011-022-03193-8**

The PDF version of this originally published article was the uncorrected proof; it has now been replaced by the corrected version.

